# Development of a model of interprofessional support interventions to enhance brace adherence in adolescents with idiopathic scoliosis: a qualitative study

**DOI:** 10.1186/s12891-022-05359-w

**Published:** 2022-04-30

**Authors:** Myriam Provost, Marie Beauséjour, Marie-Claire Ishimo, Julie Joncas, Hubert Labelle, Sylvie Le May

**Affiliations:** 1 Sainte-Justine University Health Center, Research Center, 3175 chemin de la Côte-Sainte-Catherine, Office 1.7.19, Montréal, QC H3T 1C5 Canada; 2 Department of Surgery, Université de Montréal, C.P. 6128, succursale Centre-ville, Montréal, QC H3C 3J7 Canada; 3 Department of Community Health Sciences and Centre de recherche Charles-LeMoyne-Saguenay-Lac-St-Jean sur les innovations de santé, Université de Sherbrooke, 150 Place Charles-LeMoyne – bureau 200, Longueuil, QC J4K 0A8 Canada; 4 Faculty of Nursing, Université de Montréal, 2375 chemin de la Côte-Sainte-Catherine, Montréal, QC H3T 1A8 Canada

**Keywords:** Idiopathic scoliosis, Adolescents, Brace treatment, Treatment adherence, Intervention model, Interprofessional collaboration

## Abstract

**Purpose:**

Brace treatment for adolescent idiopathic scoliosis is recognized as effective if the brace is worn as prescribed (20 to 23 hrs/day). Because of its negative biopsychosocial impact on adolescent patients’ quality of life, brace adherence is a common problem (average bracewear of 12 hrs/day). The purpose of this paper is to develop an interprofessional support intervention model to enhance brace adherence in adolescents with scoliosis.

**Methods:**

We enrolled 9 health professionals working with braced patients to participate in individual interviews. Interview guides were built following the Information-Motivation-Strategy Model (DiMatteo et al., Health Psychol Rev 6:74-91, 2012) and the Interprofessional Care Competency Framework (Education UoTCfI, Toronto Acad Health Sci Network, 2017). Thematic analysis was performed to identify the most relevant concepts for designing the intervention model. A panel of 5 clinical experts was recruited to review and validate the intervention model.

**Results:**

Participants suggested educational, motivational, functional, psychological and interprofessional teamwork strategies to improve the support provided to patients and parents and potentially increase brace adherence. Using the emerging themes and their relationships, we designed an Interprofessional Adherence Support (IPAS) intervention model that identifies the actors, activities, structure and intended impacts of the intervention. According to the expert panel, the IPAS model is highly relevant to respond to the brace adherence problem and has potential for implementation in practice.

**Conclusion:**

We designed an interprofessional support intervention model based on professional perspectives in response to the brace adherence problem in adolescents with scoliosis. Plans for implementation of the IPAS model at our scoliosis clinic are under development and considered essential for improving brace treatment outcomes.

**Supplementary Information:**

The online version contains supplementary material available at 10.1186/s12891-022-05359-w.

## Background

Brace treatment for adolescent idiopathic scoliosis (AIS) patients is believed to be the most effective conservative treatment to prevent curve progression if patients wear their brace as prescribed [1]. For full-time orthosis such as thoracolumbosacral orthosis (TLSO), a brace must be worn at least 20 to 23 hours a day, but according to a renowned international partially randomized study, North American patients wear their brace an average of only 12 hours daily [1]. Brace wear of over 12.9 hours a day was associated with a 90 to 93% chance of treatment success [1]. Brace treatment failure leads to spine correction by spinal fusion, which is more invasive and brings future physical limitations to patients. The causes of the brace adherence problem are multifactorial because bracing has an impact on patients’ functional, practical, psychological, and social well-being [2]. For example, poor body image and self-confidence, difficulties in relationships with peers, conflicts with parents at home and pain when sitting or bending the upper body have been reported in various studies [2–6]. To enhance brace adherence in AIS patients, it is recommended that interventions target more than one contributing factor through interprofessional (IP) collaboration [2, 5, 7, 8]. Dean et al. [7] reported, in a systematic review, that educational interventions alone were not sufficient to significantly enhance adherence in adolescent patients. Education paired with a behavioral or psychological component seemed to have more chances of success. Karol et al. [9] studied the effect of adherence monitoring and counseling specifically for AIS braced patients and suggested implementing such interventions in standard care. However, they reported that 14% of counseled patients were still nonadherent (< 8 h/day) [9]. This implies that personal barriers, such as psychosocial difficulties, might have been predominant for those patients and were not targeted by the intervention. Tavernaro et al. [10] found that AIS braced patients whose care was managed by a collaborative professional team (orthopedist, orthotist and physiotherapist) showed better brace adherence rates and general quality of life scores than patients managed by a standard care team. These results highlight the importance of effective and multidisciplinary teamwork for brace care management in AIS patients. However, the healthcare team professionals in these studies were mainly experts in the functional and physical aspects of bracing. Patients might still encounter adherence problems for psychosocial reasons, and support for these issues should be considered by care teams [2, 5, 6, 8, 11].

There is no biopsychosocial adherence support intervention tailored to AIS patients reported in the literature. In an effort to enhance brace adherence in AIS patients, this study aimed to develop an interprofessional adherence support intervention model tailored to healthcare providers’ perspectives, expertise, and experiences.

## Methods

### Study design

A conceptual framework analysis was used to develop the intervention model following a design method similar to the one described by Jabareen [12]. The qualitative method comprises 7 steps: mapping data sources, reading and categorizing data, identifying and naming concepts, deconstructing and categorizing concepts, integrating concepts, iteratively synthesizing the concepts and validating the conceptual framework [12].

### Setting

This study was performed at a major referral pediatric health center for spine deformities and its associated rehabilitation center.

### Sampling

For recruitment, we used purposeful and snowball sampling. The inclusion criteria were that participants were health professionals with different expertise working with AIS braced patients or braced patients with other physicallimitations. The expected sample size was 12 participants, which was estimated considering the small pool of potential participants and our need for key informants. Furthermore, we followed the maximum variation sampling type of purposeful sampling method, because we recruited professionnels of different expertise and point of view [13]. Potential participants were contacted via email using their professional correspondence information.

### Data collection

This project received ethics approval from our health center’s ethics review board. For the intervention model to reflect healthcare professionals’ scope of practice and be integrated into their care, we explored participants’ perspectives about the brace adherence problem and their ideas for strategies to support AIS patients.

We collected data through individual interviews. They lasted from 20 to 55 minutes and were carried out by teleconference on the Zoom© platform (*n* = 6) or by phone (*n* = 3). All interviews were carried out by the same interviewer (MP). The interviewer was a female M. Sc student trained in qualitative methods and wore a full-time brace herself as a teenager. She had no prior relationship with the participants and did not inform the participants of her personal experience with bracing to avoid any bias during the interview process. Informed consent for interviews to be recorded was obtained by all participants, and the recordings were deleted immediately after full transcription by the interviewer. Confidentiality was also ensured throughout the study. A semistructured guide helped structure the interviews (see Interview guide in supplementary materials). A research assistant developed the guide using two theoretical models to support the design of the intervention: the Information-Motivation-Strategy (IMS) Model by DiMatteo et al. [14] and the Interprofessional Care (IPC) competency framework designed by the Centre for Interprofessional Education at the University of Toronto [15]. The IMS Model underlines how professionals should communicate information effectively, motivate patients and provide strategies and tools for adherence [14], and it has been adapted to the specific challenges of adolescent patients dealing with chronic diseases [16]. The IPC framework identifies six domains of IP competency: patient−/family-centered care, communication, role clarity, conflict resolution, team functioning and collaborative leadership [15].

### Analyses and model design

We proceeded to a qualitative thematic analysis of the empirical data [12, 17]. A research assistant transcribed all interviews and coded transcripts deductively following concepts in the theoretical frameworks selected. New codes were also derived from the observed data in an inductive process. Validation of coding was performed by a coauthor who was blind to the recruitment and interview processes. Consensus was obtained. Code units were regrouped into broad themes, which were further broken down into subthemes in an iterative manner. Relevant themes and relationships between them were identified for inclusion in the intervention model to represent its activities, actors, timeline, purpose or intended impacts.

### Model review

The draft intervention model was shared and reviewed in a knowledge dissemination session attended by five participants with different expertise (member-checking [18]). The draft intervention model was reviewed and critiqued by 2 psychologists, 1 orthopedist, 1 clinical nurse and 1 orthotist regarding the relevance of the proposed activities and the barriers and facilitators of implementing such an intervention. A second author was present to moderate the session.

## Results

Nine professionals were enrolled: 2 orthopedists, 2 orthotists, 2 physiotherapists, 1 clinical nurse, 1 social worker and 1 psychologist. One potential nurse and 1 potential psychologist declined because they felt their field of expertise was too far from the problem studied. One potential social worker did not respond to recruitment emails. The characteristics of the participating professionals are shown in Table 1. From the interviews, seven broad themes were identified: adherence barriers; professional support barriers; educational, functional, motivational, and psychological support; and interprofessional teamwork strategies. A list of themes and associated citations are reported in Table 2. A description of the coding tree is available in the supplementary materials (see Coding tree file).

**Table 1:** Characteristics of interview participants

Participant	Gender (M/F)	Years of experience (total; with braced patients)	Place of work	Duration of interview
Orthopedist 1	M	7 years; 3.5 years	CHUSJ^a^ scoliosis clinic	25 min
Orthopedist 2	M	15 years; 15 years	CHUSJ scoliosis clinic	20 min
Orthotist 1	F	17 years; 12 years	CHUSJ scoliosis clinic	37 min
Orthotist 2	M	30 years; 24 years	CHUSJ scoliosis clinic	20 min
Physiotherapist 1	F	31 years; 31 years	CRME^b^	35 min
Physiotherapist 2	F	9 years; 9 years	CHUSJ scoliosis clinic	33 min
Clinical nurse	F	33 years; 30 years	CHUSJ scoliosis clinic	20 min
Social worker	F	35 years; 20 years	CRME	55 min
Psychologist	F	19 years; 7 years	CRME	45 min

^a^Sainte-Justine University Health Center

^b^Marie-Enfant Rehabilitation Center

**Table 2:** Interview themes and associated citations (translated from French)

Themes	Citations
Adherence barriers	• “…*there is mourning associated with the loss of self-confidence and body acceptance, especially in adolescent girls*” -Social worker • “*Often, parents suffer and we minimize their suffering. They sometimes feel guilty or responsible for their child’s scoliosis*” -Physiotherapist 1 • “*A patient may be on his own all the time or rejected*” -Clinical nurse • “*Every time parents are going through a divorce, their child’s brace treatment is at a higher risk of failure. It’s as if there were too many things going on and bracing is not a priority*” - Orthotist 1
Professional support barriers	• “*I see a lot of patients in a day, so I don’t have time to spend 30 minutes to an hour with them like a psychologist would. She* [or he] *will evaluate the family too*” -Clinical nurse • “*I sometimes would have needed to reach a social worker. To have someone in our team to directly refer families to would help*” - Orthotist 1 • “*I had parents tell me: ‘*we broke down after receiving our child’s genetic diagnosis*’. Because they were referred to us* [rehab center]*, I supported and helped them. Scoliosis patients, they aren’t referred anywhere*” -Social worker
Functional strategies	• “…*this approach of starting with a Providence* [night brace model] *and if it’s not enough, we progress to a full-time brace* […] *except if a patient’s curve leans towards surgery, we favor this approach*” -Clinical nurse • “*It’s not normal that the brace hurts patients. It might be an excuse, but they need to be reassured that their brace will be adjusted. We have to make sure that the treatment is comfortable*” -Physiotherapist 1
Educational strategies	• “…*often, people come in with preconceived ideas they found online. They see monstruous things when they do their research. I try to disarm all that by explaining that it’s not really like what they’ve seen*” -Orthopedist 2 • “*I was there to facilitate patients’ understanding by putting myself in their situation and vulgarizing medical terminology*” – Psychologist
Motivational strategies	• “*We want to encourage* [patients], *since we are their ally. Not that we’re against the parents, but we try to create a strong bond* [with patients] *since they’re wearing the brace*” - Physiotherapist 1 • “…*there should be a way for them to share. If they could hear echoes of others experiences through social groups, it would help. Socialization is very important at their age*” – Psychologist • “*I often print out two radiographs: one in-brace and one without. I sometimes give them to patients so that they look at it at home and be motivated. We easily see that the spine is straightened* [in-brace]” -Physiotherapist 2
Psychological strategies	• “*What we did is organize group sessions with a psychologist. It was interesting because patients got to know the psychologist*” -Physiotherapist 1 • “*I think that the introductive interview* [physio. and psych. dyad] *helped a lot. The psychological intervention was not in response to a difficulty; it was there as a service. It really was an integrated approach*” -Psychologist • “*Instead of making* [a nonadherent patient] *feel bad, I listened and tried to understand what was difficult at that time*” -Physiotherapist 1 • “*I often say to patients, at brace delivery, that it’s absolutely normal that they are sad, mad or that they want to throw their brace away*” -Orthotist 1 • “*I think announcing that there will be psychosocial issues throughout the bracing experience legitimizes what the patient is going through and avoids her* [or him] *from feeling abnormal*” - Psychologist
Interprofessional teamwork strategies	• “*It has always been easy to work in a way that we have access to others* […] *we work in a team format and take decisions as a team, even if I have the last word. It’s very collaborative*” - Orthopedist 2 • “*I had a great* [physiotherapist] *colleague. She believed in what I do, she gave me space and valorized interview times. She could have thought that it wasted time for physical exercises, but she understood the benefits of my work*” -Psychologist • “*Officially, patients came in for physiotherapy sessions, but we always had a global look on patients’ well-being during their brace experience* […] *We tried introducing a biopsychosocial approach from the start instead of waiting for coping issues to come up*” – Psychologist

### Adherence barriers

Under adherence barriers, we grouped together the difficulties experienced by patients undergoing brace treatment as perceived by the participating professionals. All participants mentioned at least one psychological adherence barrier, such as the initial shock of having to wear a brace, poor body image and self-confidence, stress and anxiety, and the emotional toll on parents.

The majority of participants (*n* = 6) also brought up social adherence barriers, which are believed to have an important impact on adolescents’ treatment adherence. Peer pressure, lack of professional support, and difficult family adaptation were considered significant social barriers. All participating professionals agreed that psychosocial issues are of particular concern in AIS patients going through bracing. However, they suggested that not all patients need the same level of support.

Finally, four participants mentioned functional barriers, such as the adaptation of bracing to daily activities and patient autonomy.

### Professional support barriers

The theme of professional support barriers emerged throughout the interview process and represented the difficulties or constraints participants faced when supporting patients toward better brace adherence. At an organizational level, some participants (*n* = 4) brought up the short consultation time with the orthopedist or the clinical nurse (approximately 20 minutes) allowed to each family, and one participant (*n* = 1) mentioned the 6-month interval between regular follow-up visits, which limits the extent of support professionals can provide to patients and their families.

A barrier mentioned by the participating psychologist and social worker was the biomedical perspective of treating scoliosis (prescription-based medicine), which is embedded within the organizational culture in the participating center. Concordantly, five participants mentioned the lack of psychosocial resources available in the clinic for references or assistance.

The two orthotists and one orthopedist mentioned that patients often seemed very embarrassed and did not open up about bracing issues they might have experienced. These relational barriers revealed the importance of creating and maintaining good patient-provider relationships in order to support patients on a personal level. To better address these adherence and professional support barriers, participants proposed educational, motivational, functional and psychological support strategies that they were already using in their practice or new ones that they thought could have a positive impact on brace adherence.

### Functional strategies

Participants suggested functional support strategies to improve patients’ in-brace comfort, provide physical assistance, favor a progressive brace treatment approach whenever possible, use adherence tracking methods and refer patients to external services (psychotherapy, physiotherapy, etc.).

The progressive treatment strategy was promoted by all participants working at our scoliosis clinic and consisted of prescribing a night brace to younger patients as a first-step treatment. This would allow a softer introduction of and easier coping with brace treatment before moving on to full-time bracewear if the scoliotic curve progresses. Professionals are responsible for ensuring the in-brace comfort of patients and facilitating their treatment regimen according to each patient’s prognosis.

### Educational strategies

Under educational support strategies, we grouped together the communicative approaches used by professionals to inform patients on their condition and brace treatment but also to offer ways to cope with their new situation. For example, participating physiotherapists and orthopedists described their decision-making process regarding treatment options for each patient’s specific risk factors for curve progression. Physiotherapists mentioned that they explained to patients, with supporting scientific evidence, that a brace is effective if worn appropriately. Demystifying medical knowledge and vocabulary was a strategy mentioned by orthopedists and by an orthotist as essential to clarify information found online that could be misleading or false, while the participating psychologist used this strategy to simplify medical recommendations with patients. Confirming patients’ understanding of professional recommendations was also a strategy used by the psychologist to verify patients’ comprehension of the care team’s expectations.

To cope with brace treatment, the scoliosis clinical nurse started sharing a document with patients upon brace delivery. The document highlighted important information about the treatment and tips on how to deal with it at home and at school. Participants agreed that families have to be effectively informed about the importance of wearing a brace as expected by their orthopedist.

### Motivational strategies

Motivational strategies represent the efforts undertaken by patients, their families and their friends or healthcare providers to motivate patients to adhere to brace treatment. According to most participants (*n* = 6), the establishment of a patient-provider trust relationship can be an important motivational factor for brace adherence and for following treatment guidelines. Participants (*n* = 6) also mentioned that the opportunity to be part of social groups targeting patients or parents could provide a space for sharing experiences and advice and for creating a sense of community. Support from friends and family was also a motivational factor mentioned by participants (*n* = 5), especially parents’ role in motivating patients toward treatment success at home.

These motivational strategies based on relationship quality should be promoted since adolescents tend to attribute high importance to the opinions of their peers. Empirical strategies were mentioned by participating orthopedists and physiotherapists to motivate patients by showing them actual proof of the effects of bracewear. These strategies included comparing spine curves on radiographs obtained with and without a brace, showing spine curve evolution through periodic photos and tracking actual hours in-brace.

### Psychological support strategies

Psychological support strategies represent interventions with experts and empathic approaches to help patients and their parents on a psychological level. Referral to psychological consultation was mentioned by participants (*n* = 5) as a strategy to support patients who were concerned about their mental health. Some participants (*n* = 2) agreed that group sessions with a psychologist could also be helpful for patients who need advice but are not ready for one-on-one consultations.

Other strategies proposed by the participating social worker and psychologist were more specific: offering parental support, providing access to a social worker at scoliosis diagnosis announcement and/or brace prescription, carrying out a psychological evaluation of patients and of their family dynamic and adding an introductory interview with a psychologist and a physiotherapist (or nurse) in the care pathway.

Most of these strategies are not accessible to all patients undergoing bracing, which could explain the lack of psychosocial resources that participants identified as professional support barriers. Empathic interventions are more intuitive than the interventions mentioned above and can be used by all healthcare providers. One strategy mentioned by most participants was understanding patients’ personal adherence barriers (*n* = 6). Feeling more understood can help patients feel more at ease with their healthcare provider, who in turn can support patients more effectively. Other empathic strategies included providing reassurance, normalizing emotional reactions, recognizing parents’ roles and preparing families for the potential occurrence of psychosocial issues.

These empathic communication approaches focus on patient-centered care and on the improvement of patient-provider relationships.

### Interprofessional teamwork strategies

Last, interprofessional teamwork strategies represented the actions suggested by healthcare providers to optimize teamwork and care organization. All nine participants mentioned working in close collaboration with one or more other healthcare providers for treatment decision-making, patient follow-up or any other intervention. IP coherence, role delegation in an IP team, direct communication and validation of other team members’ work were also brought up by participating professionals.

At the organizational level, three participants mentioned designating a specific healthcare provider in a coordinator role (pivot-professionnal); this team member would become a key contact person between patients and providers and would be able to direct patients to proper professionals according to their specific needs. The participating social worker and psychologist, who were accustomed to working in a rehabilitation center, proposed new strategies to incorporate into our scoliosis clinic organization, including establishing a biopsychosocial integrated approach, partnering with regional rehabilitation centers to facilitate AIS patient follow-up, partnering with rehabilitation centers to share social work services, integrating a social worker in the scoliosis clinic team and partnering with school counselors.

Some of these new strategies could be adapted and integrated into our scoliosis clinic’s care continuum to address more patient needs.

### Intervention model

**Fig. 1:**
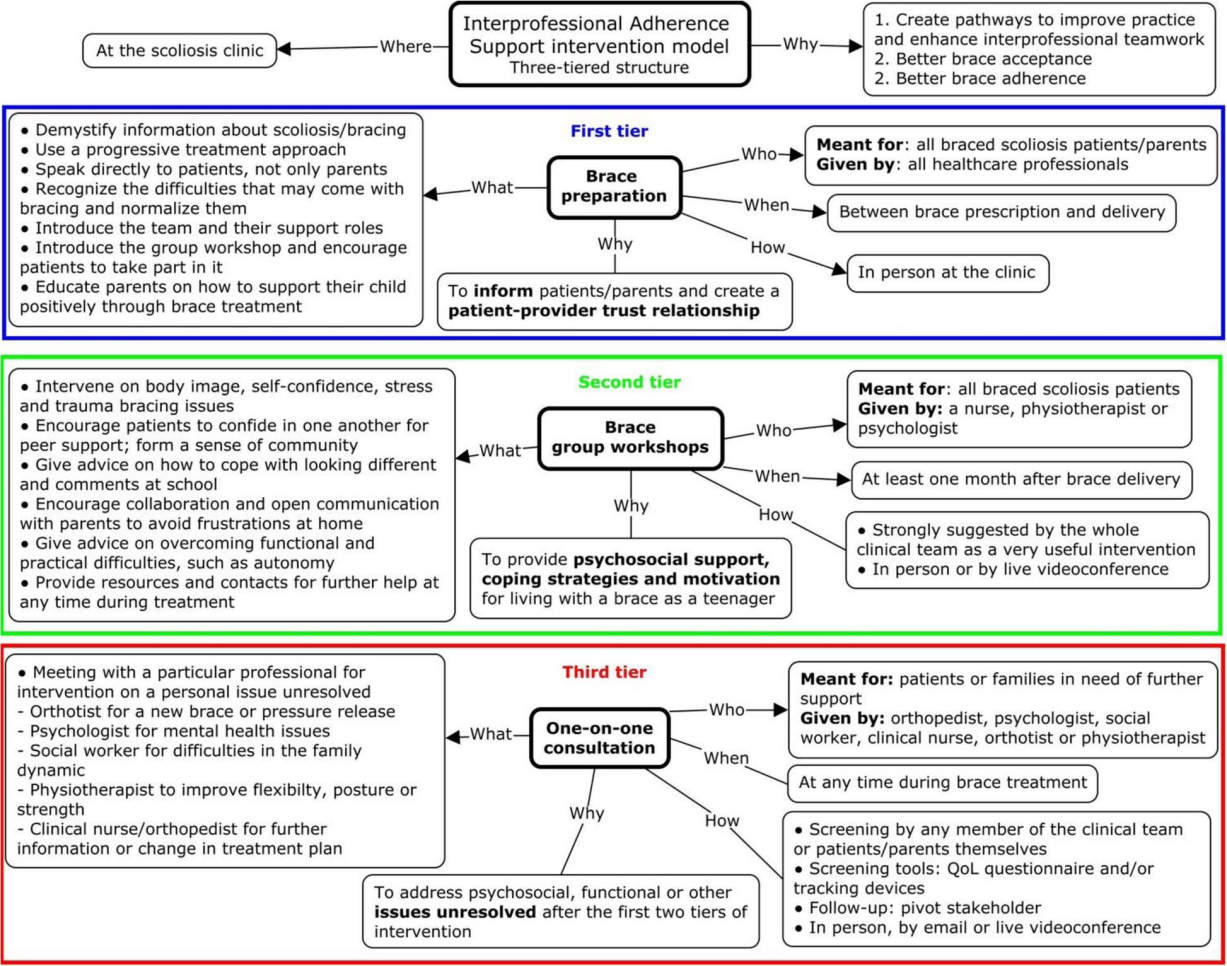
Interprofessional Adherence Support (IPAS) intervention model to enhance adherence to brace treatment in adolescents with idiopathic scoliosis

The purpose of the IPAS model (Fig. 1) is to propose a design for intervention-enhancing IP teamwork and improving patient support practices to support brace acceptance and adherence. The model is based on the premise that all patients should be given a minimum level of support and have access to additional and better targeted assistance if needed. Accordingly, the structure of the intervention is three-tiered to provide different levels of intensity of support. Each level of intervention is composed of specific components, including the purposes, involved actors and resources, activities, timing and intended impacts of the intervention.

The first tier (Fig. 1; top box) consists of preparing patients and their families for various aspects of brace treatment. Its purpose is to effectively inform families about scoliosis and bracing while engaging with patients to develop a patient-provider trust relationship.

The second tier of the IPAS intervention (Fig. 1; middle box) consists of conducting a group workshop for new brace wearers. The purpose of the workshop would be to provide psychosocial support, coping strategies and motivational strategies through discussions about body image, self-confidence, stress, and relationships at school or at home.

The third and last tier of intervention (Fig. 1; bottom box) consists of organizing one-on-one consultations between a specific professional and a patient (and parents) in need of further support to address psychosocial, functional, or other issues caused by brace treatment that are still unresolved after the two first tiers of intervention. Patients and parents can ask for further help by contacting the service stakeholder who coordinates care between families and all other team members.

### Model review

In a review of the IPAS intervention model with a panel of experts, participants agreed that the activities and purposes of the interventions were all relevant to undertaking actions to address the brace adherence issue in the adolescent scoliosis patient population. They brought up organizational barriers to the implementation of some activities, such as in standardizing the type and amount of information provided to individual patients and families and in choosing the right timing for giving group workshops in relation to brace delivery. On the other hand, they suggested important actions to be undertaken regarding implementation, including role attribution (coordinator between families and the clinical team), governance of future intervention structure and activities, adequacy of required resources and priority setting of IPAS activities.

## Discussion

The purpose of this study was to develop an interprofessional support intervention model to enhance brace adherence in AIS patients. The IPAS model is the first of its kind to address the adherence problem in the specific population of AIS patients treated with a brace. Karol et al. [9] proposed an intervention including compliance counseling and monitoring of daily brace wear hours, which has the potential to enhance brace adherence. However, their proposed intervention relies on a single support strategy and does not involve more than one professional. Our model is multileveled, involves the whole clinical team and benefits from multiple strategies to enhance adherence to brace treatment. Furthermore, the IPAS model follows the International Society on Scoliosis Orthopedic and Rehabilitation Treatment (SOSORT) recommendations regarding team management of bracing, professional commitment to compliance enhancement and patient follow-up [19]. Finally, it was built using a solid theoretical background and rigorous qualitative methods (such as consensus on coding and member checking).

Most themes yielded by the interview process were included in the IPAS model. Some were purposely excluded, such as motivation by fear of spinal surgery, collaboration with regional rehabilitation centers or collaboration with school counselors. Without intentionally trying to scare patients, a few participants mentioned that exposing the risks of spinal surgery to patients and their families was a relevant strategy that could motivate some patients to wear their brace as prescribed. However, professionals need to be cautious about using this strategy because more anxious patients could develop a deep fear regarding the possibility of going through surgery even if they wear their brace 23 hours a day. This fear could lead to lasting psychological consequences for these patients. Regarding the IP teamwork strategies mentioned, both involve many management and external resources at the scoliosis clinic. These collaboration strategies are too large scale for the purpose of this study, but this does not make them irrelevant for future developments to address the brace adherence problem.

This study had some limitations. The first was the small number of interview participants (*n* = 9), which was attributed to the fact that this project was performed at one center with a specific population of professionals. This means that we obtained partial saturation of data, which was reached for the patient adherence barriers as well as for the educational, motivational, and functional strategies’ themes. New information was provided by the participating psychologist and social worker regarding professional support barriers, psychological support and interprofessional teamwork strategies. Their contributions could be explained by the fact that they worked in a rehabilitation center with different care perspectives and had an outsider’s view of our health center’s work environment and clinical functioning. Additionally, the point of view of patients and parents was considered through findings from the literature and from the professionals’ perspectives, but patients were not active participants in the study. The IPAS model should be validated among patients before its clinical implementation is considered. Last, the IPAS model was developed to answer a treatment adherence problem for a specific population, so it is hardly generalizable (as it is presented in this paper) to the management of other diseases. However, the main interventions, such as treatment preparation, group workshops and one-on-one consultations, could be adapted to enhance treatment adherence in other populations. The IPAS model, as presented in this paper, is a three-tier intervention to enhance biopsychosocial support and potentially brace adherence. It is impossible, at this stage, to expect that it will be completely functional or effective in a clinical setting without implementing and piloting the model [20]. However, according to a study by Tavernaro et al. [10], patients who benefitted from interprofessional care management with effective collaboration between stakeholders (orthopedist, orthotist and physiotherapist) tended to be more compliant than patients treated through regular care. If all support activities and IP collaboration are carried out as planned by the IPAS model, similar results can be expected.

## Conclusions

In this qualitative study, an interprofessional intervention model was designed following input from professionals in scoliosis treatment and patient care. The IPAS model addresses a gap in the clinical functioning of our center’s scoliosis clinic and could provide improved support for patients with AIS. Since it was positively received by the panel of experts, who supported its potential to enhance adherence to brace treatment, future steps involve validation of the support activities planned among patients/parents and pilot testing of the model at our institution. An evaluation of its processes/functioning and effectiveness is also planned.

## Supplementary Information

**Additional file 1.** Interview guide.

**Additional file 2.** Coding Tree (translated from French).

## Data Availability

The datasets used and/or analyzed during the current study are available from the corresponding author on reasonable request.

## Abbreviations

AIS: Adolescent idiopathic scoliosis
IMS: Information-Motivation-Strategy model
IPC: Interprofessional Care Competency framework
IPAS: Interprofessional Adherence Support intervention model
CHUSJ: Sainte-Justine University Hospital Center
CRME: Marie-Enfant Rehabilitation Center
IP: Interprofessional
SOSORT: International Society on Scoliosis Orthopaedic and Rehabilitation Treatment
CIHR: Canadian Institutes for Health Research
