# Supplementary material for: Development of a model of interprofessional support interventions to enhance brace adherence in adolescents with idiopathic scoliosis: a qualitative study
Source: BMC Musculoskelet Disord. 2022 Apr 30;23:406. doi: 10.1186/s12891-022-05359-w (PMC9055765; doi:10.1186/s12891-022-05359-w)
Supplement: Supplementary file 1 — Additional file 1. Interview guide. [file 12891_2022_5359_MOESM1_ESM.docx]

**Interview guide**

Individual semi-structured interviews (translated from French)

1. What is your current role in the treatment care of patients with idiopathic scoliosis?
2. Through your practice, do you perceive brace nonadherence as a problem? If so, how?

- What impact does nonadherence to brace treatment have on your practice?

1. In what ways do you **inform** patients and their families about brace treatment?

- What are some of your educative strategies?
- How do you build a trusting relationship between you and your patient/family?
- How can you include patients and their families in the decision-making process regarding their treatment plan?

1. In what ways do you **motivate** patients to better follow their treatment as prescribed?
   - How do you change negative perceptions about bracing into positive ones?
   - What advice do you give parents to support their child’s brace adherence habits?
2. How do you help patients adapt to brace treatment on a day-to-day basis? What advice do you give them to keep functioning with a brace?
3. How do you collaborate with other professionals in a clinical setting? Do you think that teamwork or clinical organization could be improved?
