# Supplementary material for: Development of a model of interprofessional support interventions to enhance brace adherence in adolescents with idiopathic scoliosis: a qualitative study
Source: BMC Musculoskelet Disord. 2022 Apr 30;23:406. doi: 10.1186/s12891-022-05359-w (PMC9055765; doi:10.1186/s12891-022-05359-w)
Supplement: Supplementary file 2 — Additional file 2. Coding Tree (translated from French). [file 12891_2022_5359_MOESM2_ESM.docx]

| **Broad theme** | **Sub-theme** | **Unit code** |
| --- | --- | --- |
| **Adherence barriers**  Description:  Factors that might have a negative impact on brace adherence for patients | Psychological barriers  Description: Psychological factors that might have a negative impact on brace adherence for patients | - Body image; - Self-confidence; - Initial chock; - Parent psychological state; - Stress and anxiety; - Incomprehension of nonadherence impacts; - Feeling of unknown; - Adolescent crisis psychological impacts |
|  | Social barriers  Description: Social and relational factors that might have a negative impact on brace adherence for patients | - Family adaptation; - Lack of professional support; - Social pressure; - Social isolation; - Cultural differences; - Unstable family background |
|  | Functional barriers  Description: Functional and physical factors that might have a negative impact on brace adherence for patients | - Daily activities adaptation; - Brace autonomy; - Overloading of appointments |
| **Professional Support barriers**  Description: Factors that might have a negative impact on a professional’s capability for providing support to patients | Organization barriers  Description: Negative factors to professional support related to the organization culture or functioning | - Time-limited consultations; - 6-month follow-ups; - “Medicalized” culture of scoliosis; - Lack of continuity between hospital and rehabilitation center |
|  | Relational barriers  Description: Negative factors to professional support related to the relation between professionals and patients/families | - Silent patients - Patient/parent emotional distress; - Stigma around psychological consulting; - Non-recognition of treatment difficulty; - Sexuality issues avoided; - Coldness of Zoom consultations; - Acute surveillance of social group discussions |
|  | Referential barriers  Description: Negative factors to professional support related to the availability of external references/resources | - Accessibility of specialized physiotherapy; - Lack of psychosocial resources |
|  | Cognitive barriers  Description: Negative factors to professional support related to the development cognitive abilities of adolescents | - Immaturity of the adolescent brain; - Belief in false information |
|  | Condition-related barrier  Description: Negative factors to professional support related to scoliosis itself | - Uncertainty of the chance of curve progression |
| **Functional strategies**  Description: Strategies given by participants to enhance brace adherence on a functional level | Assistance and comfort  Description: Strategies to improve assistance from professionals or comfort in-brace | - Brace adjustment; - Materials evolution; - Heat relief; - Muscular relief by physiotherapy; - Assistance in school; - Autonomy facilitators; - Adaptation period |
|  | Progressivity  Description: Strategies to introduce brace treatment progressively in patients’ care pathway | - Progressive approach to brace treatment; - Progressive brace withdrawal; - Efficacy of night brace |
|  | Adherence tracking  Description: Strategies to improve patient awareness and avoid bias by tracking brace adherence | - Electronic tracking; - Wear level of brace |
|  | References  Description: Strategies to improve patient support by providing resources to different professionals | - Support resources |
| **Educational strategies**  Description: Strategies given by participants to enhance brace adherence on an educational level | Medical and scientific knowledge  Description: Strategies to enhance medical and scientific knowledge in patients and their families | - Decision-making process; - Demystify information; - Nonadherence risks; - Brace efficacy; - Validation of medical recommendations; - Table of risk factors; |
|  | Adaptive information  Description: Educational strategies to improve patient adaptation to brace treatment | - School presentation; - Brace model; - Info. for family and friends; - Essential info.; - Share advice and tips; - Adaptive document |
| **Motivational strategies**  Description: Strategies given by participants to enhance brace adherence on a motivational level | Relational strategies  Description: Motivation provided by family and friends | - Trust relationship; - Openness; - Family and peer support; - Parent role; - Professional supervision; - Parent social group; - Patient social group; - Mentoring |
|  | Personal strategies  Description: Motivation provided by patient himself | - Sports activities; - Belief in treatment; - Personalization of treatment; - Compromise; - Fear of curve progression/surgery |
|  | Empirical strategies  Description: Motivation provided by visual or factual proof of brace efficacy | - Periodic photos of curve progression; - In-brace radiograph; - Evolution of clinical measures; - Knowledge of adherence data |
| **Psychological strategies**  Description:  Strategies given by participants to enhance brace adherence on a psychological level | Expert interventions  Description: Psychological support strategies provided by a psychotherapist or social worker | - Presence of social worker at diagnosis reveal; - Psychological evaluation of patient; - Intake interview with physio. and psycho.; - Psychological evaluation of patient-parent relationship; - Parental support by social worker; - Group sessions with psychotherapist; - Nonadherence screening; - Reference for psychological consultation |
|  | Empathetic interventions  Description: Psychological support approaches used on a regular basis by all clinical professionals | - Understanding of adherence barriers; - Communicative approach; - Picture of clothes over brace; - Normalize reactions; - Preparing for psychosocial issues; - Parental role |
| **Interprofessional work strategies**  Description: Strategies given by participants to improve collaboration in a clinical or external setting | Teamwork strategies  Description: Strategies to improve teamwork between individuals | - Interprofessional collaboration; - Interprofessional coherence; - Direct communication; - Roles in IP team; - Recognize others work value |
|  | Organization strategies  Description: Strategies to improve collaboration through organizational approaches | - Link/pivot; - Integrated psychosocial approach; - Integrated social worker; - Connection with regional rehab. centers; - Partnership with Marie-Enfant rehab center; - Partnership with school counselors |
